# Peer-to-peer training among health care professionals working in dialysis clinics: an education approach from the GoodRENal project

**DOI:** 10.1007/s40620-024-02074-8

**Published:** 2024-10-01

**Authors:** Carla Maria Avesani, Alicia García-Testal, Patricia Mesa-Gresa, Alexandra-Elena Marin, Amaryllis H. Van Craenenbroeck, Evangelia Kouidi, Naomi Clyne, Eva Segura-Ortí

**Affiliations:** 1 Division of Renal Medicine and Baxter Novum, Department of Clinical Science, Technology and Intervention, Karolinska Institute, M99 Karolinska University Hospital, Stockholm, Sweden; 2 Nephrology, Hospital de Manises, Manises, Spain; 3 Psychobiology Department, Psychology and Logopedia Faculty, Universitat de València, Valencia, Spain; 4 Department of Nephrology, University Hospitals Leuven, Louvain, Belgium; 5 Laboratory of Sports Medicine, Aristotle University of Thessaloniki, Thessaloniki, Greece; 6 Department of Nephrology, Clinical Sciences Lund, Skåne University Hospital and Lund University, Lund, Sweden; 7 Physiotherapy, Universidad Cardenal Herrera-CEU, CEU Universities, Alfara del Patriarca, Spain; 8 Department of Microbiology, Immunology and Transplantation, Nephrology and Renal Transplantation Research Group, KU Leuven, Louvain, Belgium

**Keywords:** Chronic kidney disease, Hemodialysis, Health care professionals, Nutrition, Physical activity, Emotional well-being

## Abstract

**Background:**

Lifestyle interventions aiming to improve dietary habits, increase physical activity level, and improve emotional well-being can positively impact clinical outcomes in patients with chronic kidney disease (CKD). Educational material for health care professionals working with CKD patients that focuses on why and how to promote lifestyle changes is lacking. The present study aims to depict the material and dissemination methods for the peer-to-peer training program developed for health care professionals working in the dialysis clinics of the four countries engaged in the GoodRENal project: Spain, Greece, Sweden, and Belgium.

**Methods:**

This is an ERASMUS + project funded by the European Union (number 2020–1-ES01-KA2014-083141, http://goodrenal.eu/) named GoodRENal. The educational material was developed in English by a multidisciplinary team integrating the GoodRENal project (dietitian, physiotherapist, psychologist, and nephrologist). The material was then translated to Greek, Spanish, Swedish and Dutch and is available for download at the GoodRENal webpage (https://goodrenal.es/results-3/). After training, the health care professionals filled in an anonymous questionnaire regarding their degree of satisfaction with the training.

**Results:**

In total, 138 health care professionals in the four dialysis clinics joined the peer-to-peer training, representing 50% to 92% of the health care professionals in each clinic. From the total sample, 78 health care professionals responded to the satisfaction questionnaire. The answers showed that most participants were very satisfied or satisfied with the peer-to-peer training and that they found this approach useful in their clinical practice.

**Conclusion:**

The educational material developed for health care professionals working with patients on hemodialysis (HD) obtained good satisfaction scores from the participants.

**Graphical Abstract:**

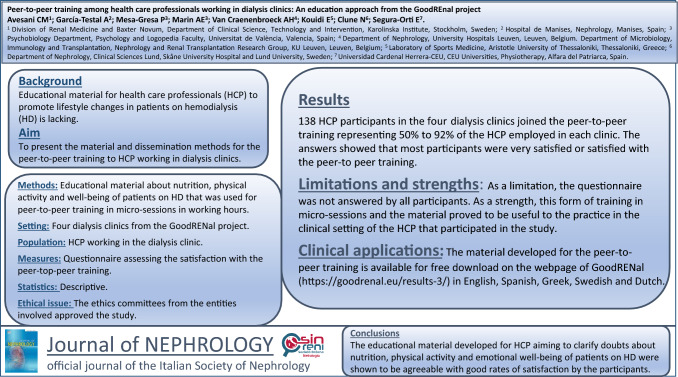

## Introduction

Lifestyle interventions aiming to improve dietary habits, to increase physical activity level and to improve emotional well-being are considered to positively impact clinical outcomes in patients with chronic kidney disease (CKD) in all stages of the disease. These clinical outcomes include improvement in body weight control, blood glucose, blood pressure and overall quality of life (QoL), in addition to improving survival rates [1–3]. However, implementing lifestyle changes is not always easy [4]. Among the factors that can improve motivation are the involvement of carers and health care professionals (HCP) with the patient, and the development of educational material clarifying why and how to promote changes that are aligned with priorities depending on specificities related to the patient´s clinical condition and possibilities to implement these changes [5]. Because the disease itself and the dialysis treatments are quite complex, patients, carers and health care professionals often lack knowledge of aspects related to lifestyle. These include the specific foods to choose that could avoid the accumulation of certain minerals, electrolytes, hormones, and ions that are hazardous to a patient’s health either in the short term or long term; how to adopt a physically active lifestyle and which physical exercises and/or exercise program to follow; how to be more physically active without risking the occurrence of cardiovascular events or other comorbidities, and finally, how to deal with factors related to emotional well-being and cognitive state that often can decrease one’s overall disposition to take care of themselves. Within this scenario, CKD-related guidelines have highlighted the need to develop strategies that can provide continuous education to the stakeholders involved in the treatment – i.e., patients, carers and health care professionals [6–9]. Under this concept, the GoodRENal project was created with three aims in mind: 1. to create educational material for, and to educate health care professionals, 2. to create educational material for, and to educate patients on hemodialysis (HD) and their carers, 3. to create a digital platform with a program aimed at providing information on intradialytic patient education and physical training during HD, with regard to nutrition, physical activity, cognition and psychological well-being. The aim of the current study, presented as a technical report, is to present the material and dissemination methods for the peer-to-peer training developed for health care professionals working in the dialysis clinics of the four countries engaged in the GoodRENal project: Spain, Greece, Sweden and Belgium. In addition, the satisfaction rating of the health care professionals with the education training was evaluated.

## Methods

This is an ERASMUS + project funded by the European Union (number 2020–1-ES01-KA2014-083141, http://goodrenal.eu/) named GoodRENal that aims to develop educational material for patients on HD, their carers and health care professionals working in the dialysis clinics of four countries: 1. Renal Unit of AHEPA Hospital, Thessaloniki, Greece; 2. Hospital de Manises, Nephrology Service, Manises, Spain; 3. Skåne University Hospital, Department of Nephrology, Lund, Sweden and, 4. University Hospitals Leuven, Department of Nephrology, Leuven, Belgium. As part of this project, educational material was developed based on issues raised by the patients, health care professionals and carers in exploratory questionnaires previously administered based on what they perceived as their main needs, barriers and facilitators to enable adherence to dietary recommendations, increase physical activity and improve emotional well-being [10]. The educational material was developed by a group of clinicians comprising 3 nephrologists, 3 physiotherapists, 2 psychologists and one dietitian. The physiotherapists and the dietitian are trained professionals that work with specificities inherent to CKD and dialysis treatment. The material was developed aiming to discuss five main drivers using the following questions: Why is it important?, Who are the candidates?, When and where?, What and how?, and Which are the alarm signals?. This educational material aimed to provide content for the peer-to-peer-training of health care professionals from the participating dialysis units about specific aspects related to physical activity, adherence to nutrition recommendations, cognitive state and psychological well-being of patients on chronic HD. All health care professionals working in the dialysis unit were invited to participate in the peer-to-peer sessions. The group that participated in the training sessions varied from one center to another, but in general included the nephrologists, nurses, dietitians and physiotherapists. The educational material comprised three brochures, one for Nutrition, a second for physical activity, and a third for psychological and cognitive well-being. Training took place between March and October, 2022 at the dialysis clinics of each participating center, as shown in Table 1. It was adapted according to staff availability and to the room in which the training was carried out. As most HD units suffer from a shortage of staff and staff with busy schedules, a novel methodology was developed consisting of micro-sessions of training. These micro-sessions took place at a work station during their regular activities within the HD unit. The sessions were led either by a physiotherapist or by a nurse responsible for educating staff on the basis of the availability of each HD unit. The material in the brochures was developed in English and then translated to Greek, Spanish, Swedish and Dutch by native speakers from each country. This translated material can be found at the GoodRENal webpage (https://goodrenal.es/results-3/). From 1 to 30 days after the end of training, the participants were asked to anonymously respond to an electronic survey on the *User Satisfaction Evaluation Questionnaire* with 10 statements about their opinion on the peer-to-peer training. The answers for the statements were rated from 1 to 5 as very satisfied, satisfied, neutral, unsatisfied, and very unsatisfied, respectively, and from statements 6 to 10 as strongly agree, agree, neutral, disagree, and strongly disagree, respectively. This study complies with the criteria of the Declaration of Helsinki. The ethics committees from the entities involved approved the study. Since the current study has no intervention nor processing of personal data in the electronic questionnaire, there was no need for the participants to sign an informed consent form prior to their participation in the study.

**Table 1:** Description of the peer-to-peer training in the dialysis clinic according to the participating dialysis center

	Number of participants/ HCP employed	Number of sessions/ duration (min)/ HCP participated in the training	Material used	HCP leading the peer-to-peer training	Online or Face-to-face
Thessaloniki, Greece	32/40	2/30 min for nurses 1/40 min for nephrologists 1/40 min for dietitians	Flashcards and PPT presentation	Nurse, nephrologist	Face-to-face Online Online
Manises, Spain	39/45	30 sessions 40 min Nephrologist, nurse	PPT presentation	Nurse, nephrologist	Face-to-face
Lund, Sweden	37 /44	3/30 min for nurses 1/40 min for nephrologists	Flashcards and PPT presentation	Physiotherapist, nurse and nephrologist	Face-to-face Face-to-face
Leuven, Belgium	30/61	15/10 min Nephrologist, nurse, dietitian	Flashcards and PPT	Nurses and dietitians	Face-to-face

*HCP* health care providers; *PPT* power point

## Results

The content of the educational material is summarized in Table 2 (Physical activity), Table 3 (Nutrition) and Table 4 (Cognition and Psychological well-being), but each center adapted the material to be used in their own dialysis center. The idea was to give a practical overview so that the health care professional could be an ambassador of good practices regarding physical activity, nutrition, cognition, and psychological well-being and then refer the patient to receive specific attention from the corresponding health care professional in the clinic. During the peer-to-peer training, there were 32 health care professional participants in the dialysis clinic in Thessaloniki (12 on-site and 20 online), 39 health care professionals in Manises (on-site), 37 in Lund (on-site) and 30 participants in Leuven (on-site) (138 participants altogether). The participants represent 50% to 92% of the health care professionals employed in each clinic (Table 1). After the training, the health care professionals were invited to respond to an anonymous questionnaire regarding their satisfaction with the training (Table 5). From Manises, 24 out of 39 participants replied to the *User Satisfaction Evaluation Questionnaire*, in Thessaloniki all participants replied (*n* = 32), in Lund due to a technical issue, the initial survey vanished and a second survey was sent 2 months later to the participants, of whom 11 out of 37 replied, and in Leuven 11 out of 30 replied to the questionnaire (a total of 78 responded the questionnaire). The answers showed that most participants were very satisfied or satisfied with the peer-to-peer training. In addition, most of the answers strongly agreed or agreed that the peer-to-peer training was useful for their practice in the clinical setting. In Sweden the educators reported a very positive response from the health care professionals involved; they were eager to participate and in the framework of a small informal group felt free to ask questions and discuss.

**Table 2:**
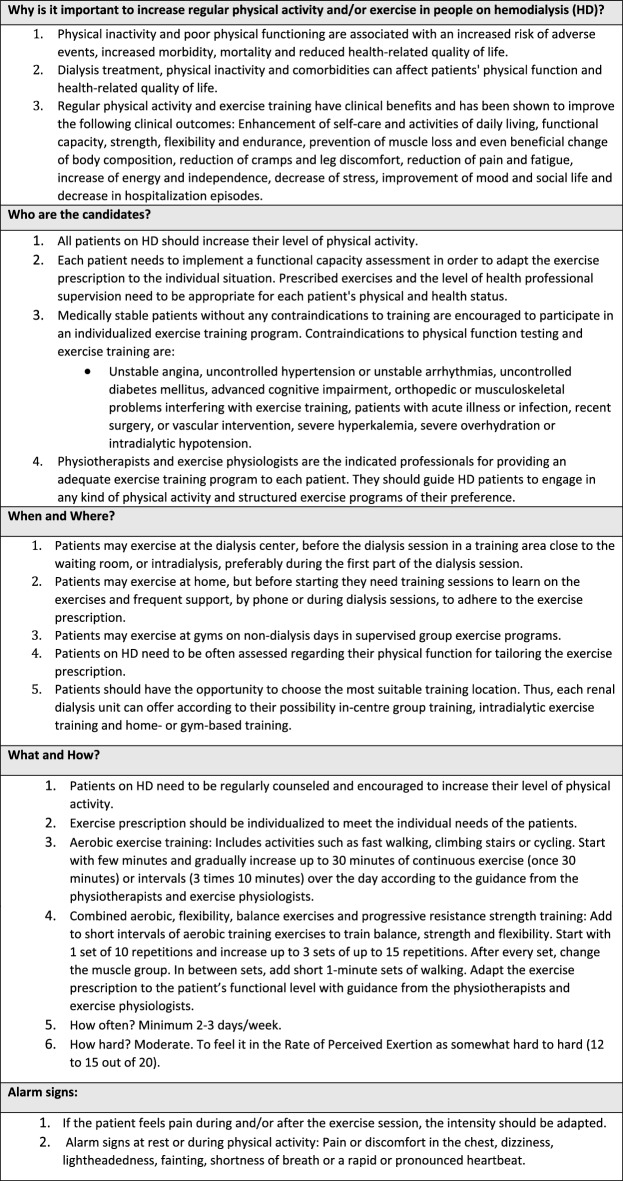
Recommendations regarding physical activity and exercise to people on hemodialysis in order to improve general health and well-being

**Table 3:**
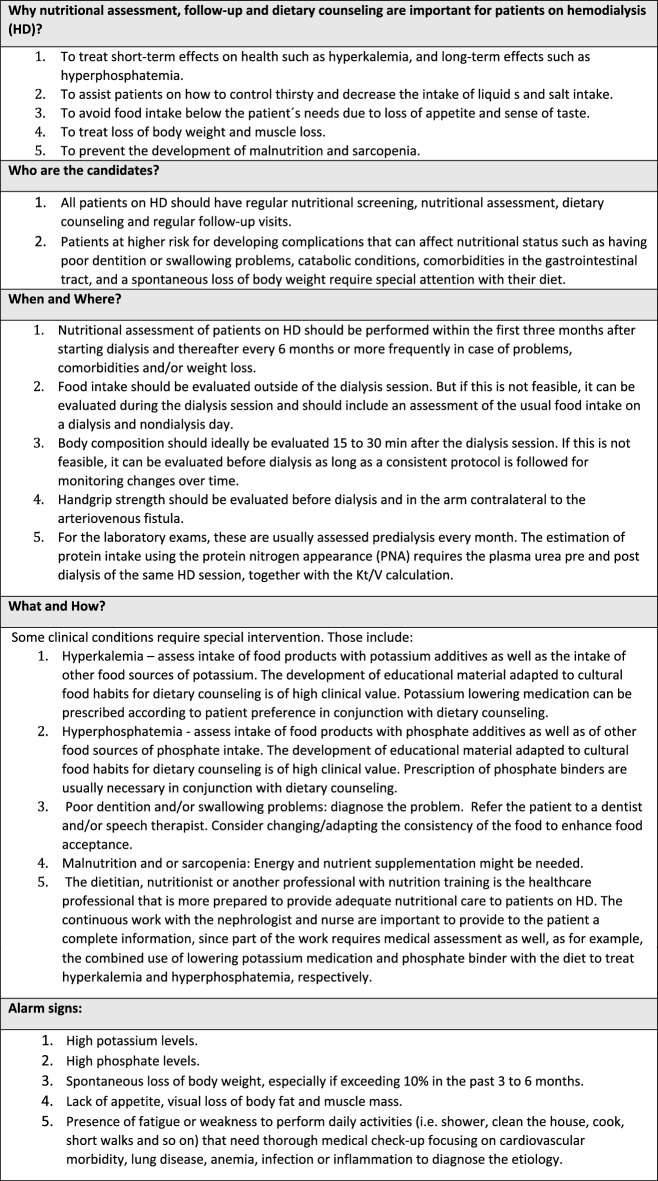
Recommendations regarding nutrition to people on hemodialysis in order to improve dietary adherence

**Table 4:**
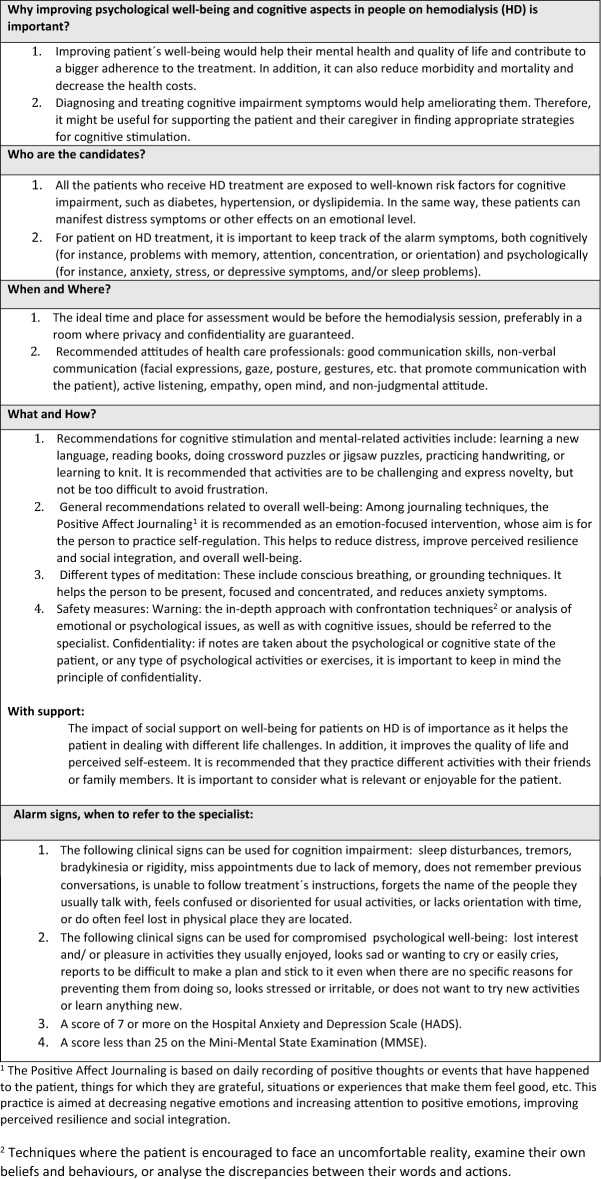
Recommendations regarding psychological and cognitive well-being to people on hemodialysis

**Table 5:** Results from the peer-to-peer training on aspects related with physical activity, nutrition, cognition and psychological well-being of patients on chronic HD as reported by health care professionals working in dialysis clinics (*n* = 78)

Statements	Answers				
	Very satisfied	Satisfied	Neutral	Unsatisfied	Very unsatisfied
Q1. How satisfied were you with the peer-to-peer training?					
Thessaloniki (*n* = 32)	87.50%	12.50%	–	–	–
Manises (*n* = 24)	56%	36%	8%	–	–
Lund (*n* = 11)	27.30%	54.50%	18.20%	–	–
Leuven (*n* = 11)	37%	54%	9%	–	–
Q2. How satisfied were you with your participation in the themes discussed in the peer-to-peer training?					
Thessaloniki (*n* = 32)	78.10%	21.90%	–	–	–
Manises (*n* = 24)	28%	60%	8%	4%	–
Lund (*n* = 11)	27.30%	54.50%	18.20%	–	–
Leuven (*n* = 11)	–	64%	36%	–	–
Q3. Were you satisfied with the number of participants in the peer-to-peer training?					
Thessaloniki (*n* = 32)	75%	25%	–	–	–
Manises (*n* = 24)	36%	48%	12%	4%	–
Lund (*n* = 11)	45.4	27.30%	27.30%	–	–
Leuven (*n* = 11)	27.30%	54.50%	18.20%	–	–
Q4. Were you satisfied with the time allocated to the peer-to-peer training?					
Thessaloniki (*n* = 32)	90.60%	9.40%	–	–	–
Manises (*n* = 24)	28%	40%	28%	4%	–
Lund (*n* = 11)	9.10%	54.50%	27.30%	9.10%	–
Leuven (*n* = 11)	18.20%	45.40%	18.20%	18.20%	–
Q5. Were you satisfied with the support material used for this learning activity?					
Thessaloniki (*n* = 32)	78.10%	21.90%	–	–	–
Manises (*n* = 24)	24%	64%	12%	–	–
Lund (*n* = 11)	27.30%	72.70%	–	–	–
Leuven (*n* = 11)	18.20%	45.40%	27.30%	9.10%	–
Q6. If you felt uncomfortable during the peer-to-peer training, please indicate the reasons					
Thessaloniki (*n* = 32) Manises (*n* = 24) Lund (*n* = 11) Leuven (*n* = 11)	No comments				

	Strongly agree	Agree	Neutral	Disagree	Strongly disagree
Q7. The information provided in the peer-to-peer training was clear					
Thessaloniki (*n* = 32)	90.60%	9.40%	–	–	–
Manises (*n* = 24)	50.60%	49.40%	–	–	–
Lund (*n* = 11)	45.50%	45.50%	9%	–	–
Leuven (*n* = 11)	27.30%	72.70%	–	–	–
Q8. The information provided in the peer-to-peer training was easy to understand					
Thessaloniki (*n* = 32)	87.50%	12.50%	–	–	–
Manises (*n* = 24)	64%	36%	–	–	–
Lund (*n* = 11)	55.50%	45.50%	–	–	–
Leuven (*n* = 11)	27.30%	63.80%	9.10%	–	–
Q9. I was successful in learning what was discussed in the peer-to-peer training					
Thessaloniki (n = 32)	81.30%	18.70%	–	–	–
Manises (*n* = 24)	56%	44%	–	–	–
Lund (*n* = 11)	45.50%	45.50%	9%	–	–
Leuven (*n* = 11)	18.20%	72.70%	9.10%	–	–
Q10. The information provided in the peer-to peer training is applicable to my clinical setting					
Thessaloniki (*n* = 32)	90.60%	9.40%	–	–	–
Manises (*n* = 24)	48%	52%	–	–	–
Lund (*n* = 11)	27.30%	63.60%	9.10%	–	–
Leuven (*n* = 11)	–	81.80%	18.20%	–	–

## Discussion

GoodRENal is an educational ERASMUS + project aiming to develop educational material to improve adherence to dietary recommendations, increase physical activity and improve cognitive and emotional well-being of patients on HD. In order to do that, educational material related to these 3 arms were developed not only for patients, but also for carers and health care professionals to cover the 3 stakeholders involved in the treatment of patients on HD. This technical report shows that the material developed in the form of peer-to-peer training was of interest to health care professionals working in HD clinics. The material we developed includes nutrition, physical activity, cognition, and emotional well-being. The issues chosen to be discussed were those raised by the health care professionals in exploratory questionnaires that are part of the GoodRENal project [10]. This approach allowed the stakeholders to shape the priorities to achieve positive changes. We believe that this methodology can explain the high rate of satisfaction with the peer-to-peer training, since most participants rated their experience as “very satisfied” or “satisfied”. Of note, when the statement investigated their perception of whether “the material used was applicable to their clinical setting”, 100% to 81% of the participants replied “strongly agree” or “agree” with the statement. This finding suggests that the information circulated in the peer-to-peer training will likely be useful to the health care professionals in their daily clinical practice.

Guidelines for the care of CKD patients advise using a model of care that includes multidisciplinary education for health care professionals working with patients with CKD [6–9]. As is well described in guidelines for CKD, adhering to lifestyle changes such as diet and modified physical activity requires a combined effort by patients and carers with support from health care professionals [6–9]. Acquiring knowledge on the reason for promoting lifestyle changes, as well as on how to achieve and incorporate them into everyday life is important. Patients, carers and health care professionals do have many doubts on what the risks regarding food and physical activity are, yet not much effort has been made to provide information to clarify their doubts. Providing training to health care professionals is part of an accessible and equitable effort to shape positive changes in patients, as recommended in the CKD and Diabetes guidelines [6, 7]. The KDOQI Nutrition Guidelines also reinforce the importance of continuous educational training for dietitians in planning medical educational therapy for patients on dialysis [8]. Finally, the approach of peer-to-peer training highlights that no one holds all knowledge, on the contrary, the lively discussion mediated by a facilitator with the use of educational material allows active participation, an increased degree of interest and greater knowledge retention. We believe that the materials that have been developed can be of use to other dialysis centers. A limitation may be that there were no nurses involved in the development of the educational material for the peer-to-peer training. Since nurses are the health care professionals who spend the greatest amount of time with dialysis patients, their participation would likely have improved the quality of the developed material. Second, the questionnaire assessing the impression of the peer-to-peer training was not filled in by all participants. A strength of the study is that this form of training, carried out in micro-sessions, was adaptable to the routine of the dialysis clinics, and the material was useful in the clinical setting of the health care professionals that participated in the study.

In conclusion, the educational material developed for health care professionals, aimed at clarifying doubts about the nutrition, physical activity and emotional well-being of patients on HD, proved to be agreeable with good rates of satisfaction by the participants. All the material developed for this activity is available for free download on the webpage of GoodRENal (https://goodrenal.es/results-3/) in English, Spanish, Greek, Dutch and Swedish. We hope that the educational material and dissemination method can be of use to other centers within the context of a combined effort to achieve positive changes in lifestyle behavior by increasing adherence to dietary recommendations, physical activity, and amelioration of emotional well-being.

## Data Availability

Data can be made available upon reasonable request.
